# Supplementary material for: H3K4me2 distinguishes a distinct class of enhancers during the maternal-to-zygotic transition
Source: bioRxiv. 2024 Aug 26:2024.08.26.609713. Preprint. [Version 1] doi: 10.1101/2024.08.26.609713 (PMC11383010; doi:10.1101/2024.08.26.609713)
Supplement: 1 [file NIHPP2024.08.26.609713V1-supplement-1.pdf]

bioRxiv preprint doi: https://doi.org/10.1101/2024.08.26.609713; this version posted August 26, 2024. The copyright holder for this preprint (which was not certified by peer review) is the author/funder, who has granted bioRxiv a license to display the preprint in perpetuity. It is made available under aCC-BY 4.0 International license.

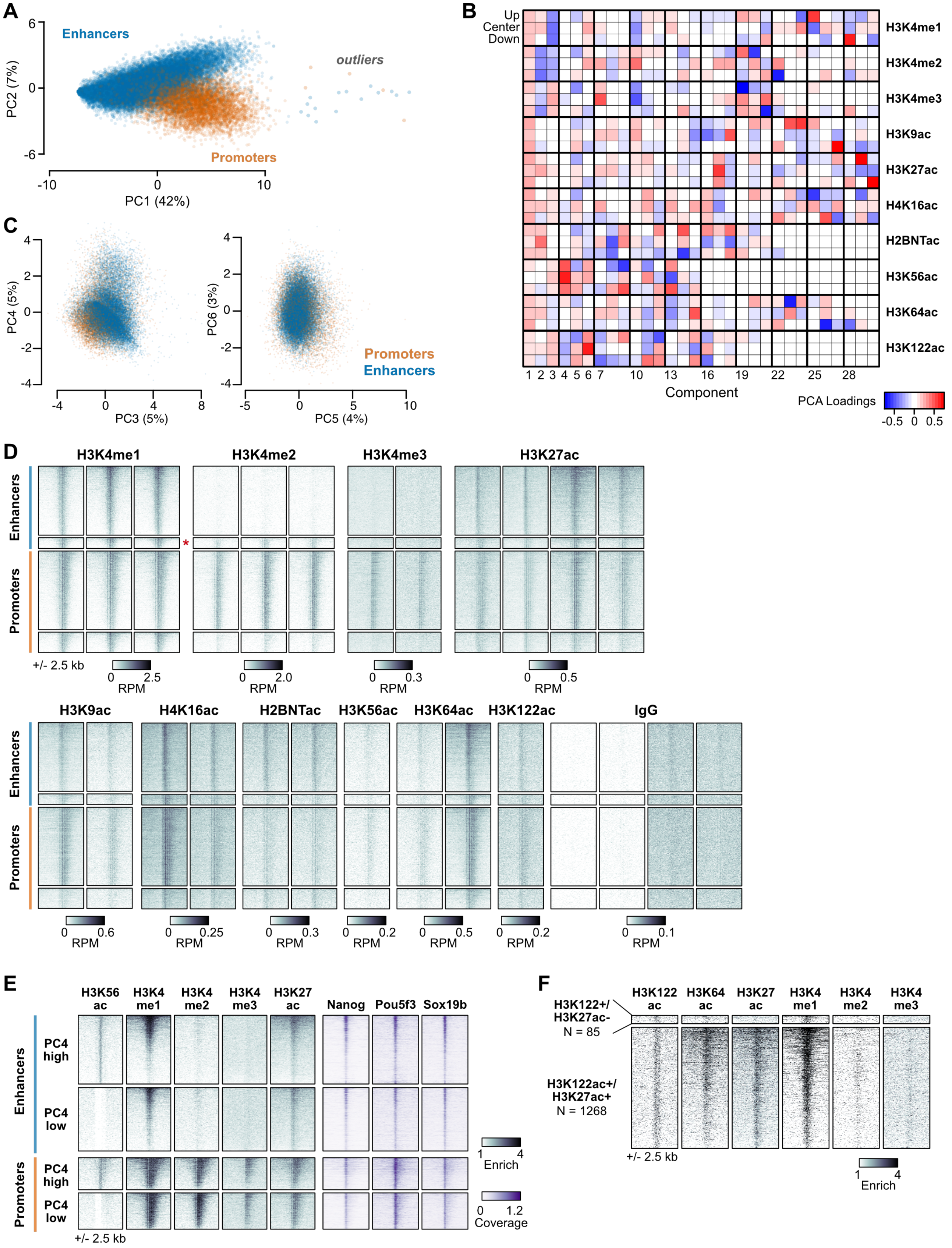

bioRxiv preprint doi: https://doi.org/10.1101/2024.08.26.609713; this version posted August 26, 2024. The copyright holder for this preprint (which was not certified by peer review) is the author/funder, who has granted bioRxiv a license to display the preprint in perpetuity. It is made available under aCC-BY 4.0 International license.

Supplementary Figure 1. Principal component analysis on histone modifications. (A) Full biplot of the first two principal components (PCs) as in Fig. 1D, including outliers far from the main point masses. Percent of total variance explained per PC in parentheses. Points are labeled as enhancers (blue) or promoters (orange). **(B)** Heatmap of the loadings from the PCA. Columns are principal components, rows are input variables – histone modification coverage on upstream, center, and downstream regions of predicted regulatory elements. **(C)** Biplots as in (A) for PCs 3 through 6. **(D)** Heatmaps of CUT&RUN coverage as in Fig 1F showing individual replicates. **(E)** Heatmaps of regions stratified by the fourth PC, which loads heavily on H3K56ac. PC4 high = greater than the standard deviation of PC4, PC4 low = less than -1 * standard deviation of PC4. Parallel heatmaps of Nanog, Pou5f3, and Sox19b ChIP-seq coverage (data from Miao et al, 2022) demonstrate minimal differences correlated with PC4, in contrast to mouse ES cells in which Pou5f3 homolog Oct4 correlates with H3K56ac (Tan et al, 2013). **(F)** Heatmaps of regions enriched for H3K122ac (>2-fold over IgG), stratified by H3K27ac co-enrichment (<1.25-fold or >1.5-fold).

bioRxiv preprint doi: https://doi.org/10.1101/2024.08.26.609713; this version posted August 26, 2024. The copyright holder for this preprint (which was not certified by peer review) is the author/funder, who has granted bioRxiv a license to display the preprint in perpetuity. It is made available under aCC-BY 4.0 International license.

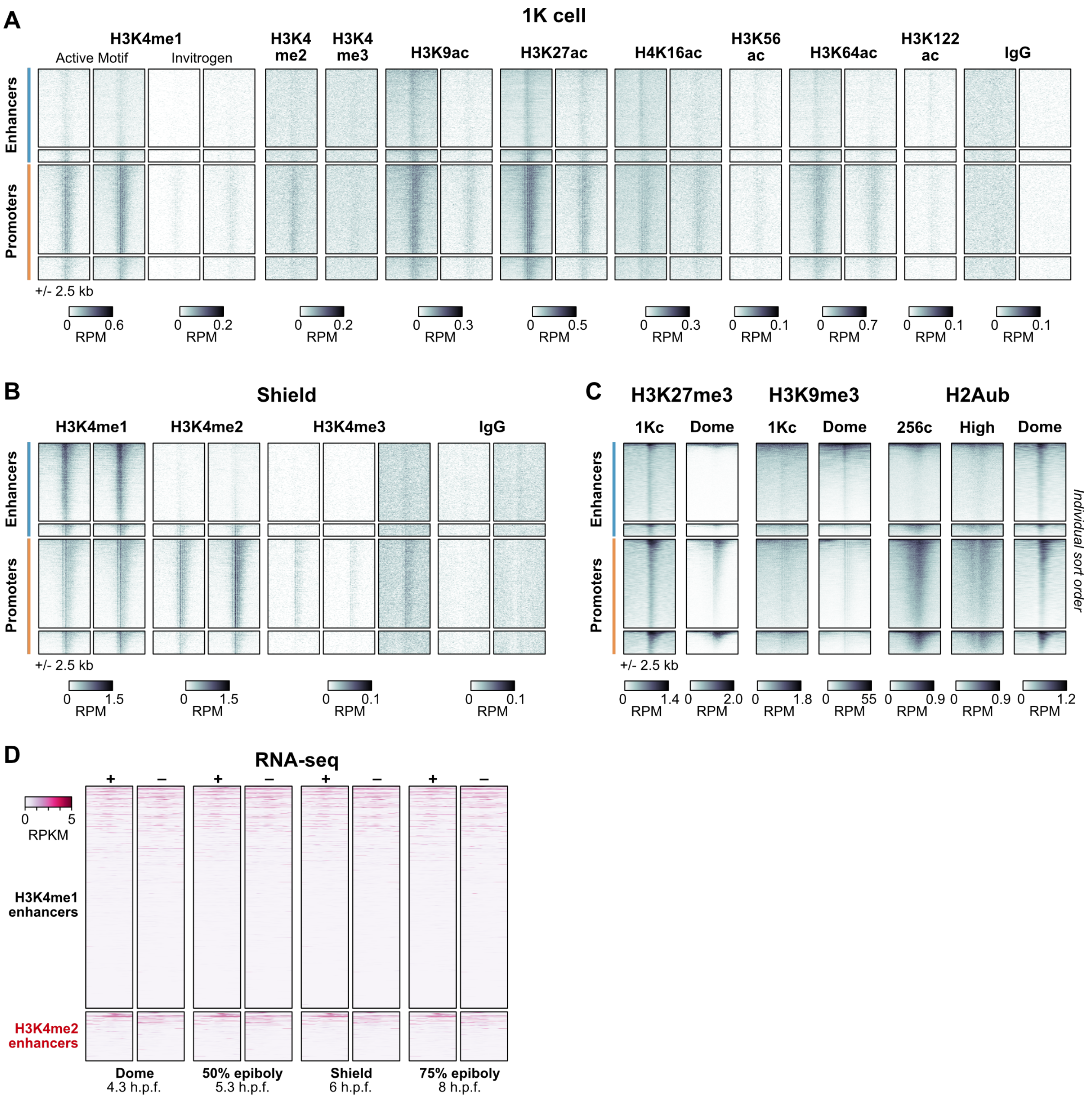

Supplementary Figure 2. Genomic profiles over time. (A) Heatmaps of CUT&RUN coverage for histone modifications at 1K-cell stage centered on enhancer and promoter regions as defined in Fig. 1F. Individual replicates are shown. Two different H3K4me1 antibodies were used, Active Motif #39297 and Invitrogen #710795 (the same antibody used for all other time points). **(B)** Heatmaps of CUT&RUN coverage at shield stage showing individual replicates. **(C)** Heatmaps of ChIP-seq for repressive histone modifications. Each heatmap is sorted by descending signal per region group independently. Data are from Zhu et al, 2019 (1K-cell H3K27me3), Zhang et al, 2014 (dome H3K27me3), Duval et al, 2024 (H3K9me3), and Hickey et al, 2022 (H2Aub). **(D)** Strand-separated RNA-seq coverage heatmaps as in Fig. 2B showing intermediate developmental stages. Data are from White et al, 2017.

bioRxiv preprint doi: https://doi.org/10.1101/2024.08.26.609713; this version posted August 26, 2024. The copyright holder for this preprint (which was not certified by peer review) is the author/funder, who has granted bioRxiv a license to display the preprint in perpetuity. It is made available under aCC-BY 4.0 International license.

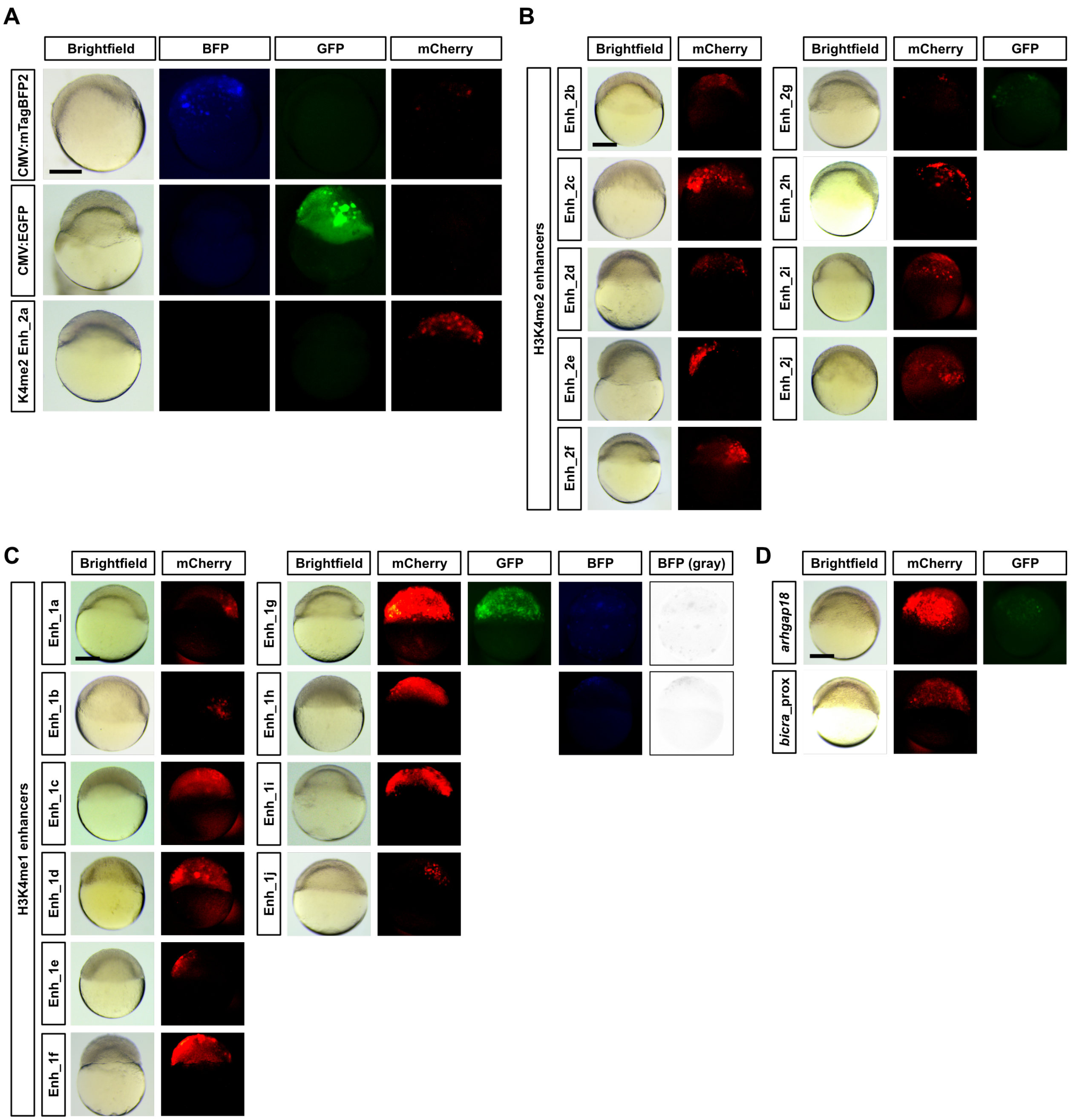

Supplementary Figure 3. Reporter assays for regulatory elements. (A) Representative embryos injected with reporter plasmids (top to bottom, CMV promoter oriented toward the mTagBFP2, CMV promoter oriented toward the EGFP, and H3K4me2 reporter Enh_2a) imaged in brightfield and BFP, GFP, and mCherry channels. Counts are reported in Supplementary Table X. **(B, C)** Representative embryo fluorescence for additional mCherry-positive enhancers. Panels for reporters additionally yielding GFP and/or BFP fluorescence are also shown. BFP fluorescence in these embryos was weak, so grayscale versions of the BFP images are also shown. Enhancers Enh_2i and Enh_2j are the *ier5l* enhancers tested in the CRISPR-Cas9 experiments. **(D)** Representative embryos for a promoter (*arhgap18*) reporter and an element proximal (< 2kb) to the *bicra* TSS, both showing mCherry fluorescence. Scale bar = 250 µm.

bioRxiv preprint doi: https://doi.org/10.1101/2024.08.26.609713; this version posted August 26, 2024. The copyright holder for this preprint (which was not certified by peer review) is the author/funder, who has granted bioRxiv a license to display the preprint in perpetuity. It is made available under aCC-BY 4.0 International license.

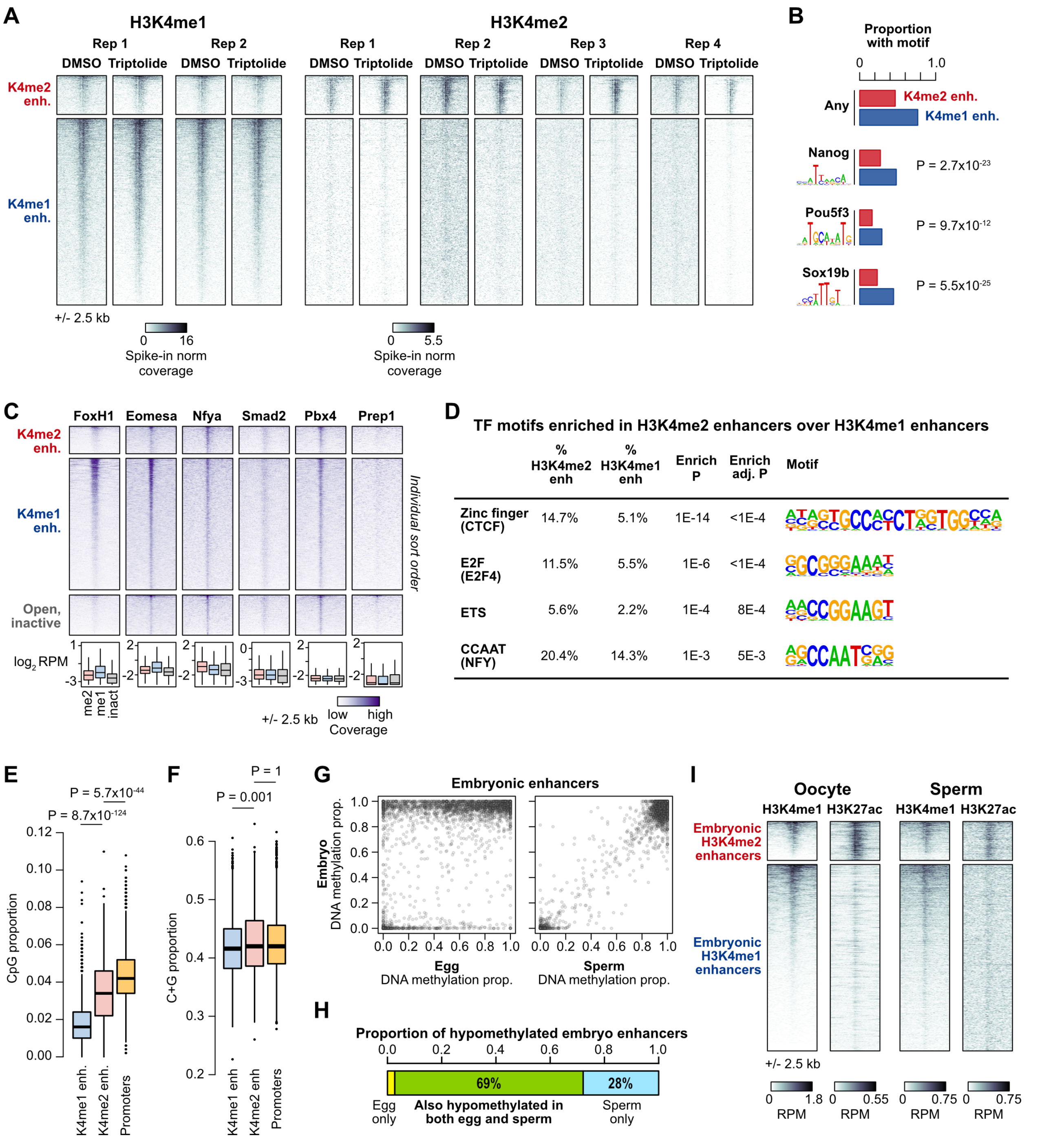

Supplementary Figure 4. Chromatin characteristics of the two enhancer classes. (A) CUT&RUN heatmaps as in Fig. 4A showing individual replicates. Each replicate is a paired DMSO control and triptolide treatment group. **(B)** Barplots showing proportion of enhancers containing predicted zebrafish Nanog, Pou5f3, and Sox19b binding sequences as represented by sequence logos (left) empirically determined from ChIP-seq (data from Miao et al, 2022). P values for Chi-squared tests (2 d.o.f.) are listed on the right. (cont'd...)

bioRxiv preprint doi: https://doi.org/10.1101/2024.08.26.609713; this version posted August 26, 2024. The copyright holder for this preprint (which was not certified by peer review) is the author/funder, who has granted bioRxiv a license to display the preprint in perpetuity. It is made available under aCC-BY 4.0 International license.

(cont'd) **(C)** Heatmaps showing ChIP-seq coverage for different embryonic transcription factors on enhancer regions as well as 1000 ATAC-seq open regions lacking enrichment for any dome-stage histone modifications. Each heatmap is individually sorted in descending order per group. Boxplots summarizing coverage are below each heatmap (boxes are first through third quartiles, center bar median, whiskers extend to 1.5x the interquartile range, outliers are not shown). Data are from Dubrulle et al, 2015 (dome stage FoxH1 and Smad2), Miao et al, 2022 (sphere stage Nfya and Eomesa), Ladam et al, 2018 (high/oblong stage Prep1), Stanney et al, 2020 (high/oblong-stage Pbx4). **(D)** Table of top enriched transcription factor binding motifs in H3K4me2 enhancers relative to H3K4me1 enhancers. One representative motif per family is shown. **(E, F)** Boxplots showing CG dinucleotide (CpG) and C+G nucleotide prevalence in 500 bp centered on H3K4me1 enhancers, H3K4me2 enhancers, and active TSSs. P-values for Wilcoxon rank sum tests are shown. **(G)** Biplots comparing DNA methylation proportion in gametes (x axes) versus sphere stage embryos (y axes) for predicted enhancers. **(H)** Stacked barplot showing the proportion of hypomethylated (<20% methylated) embryonic enhancers that are also hypomethylated in gametes. **(I)** Gamete H3K4me1 and H3K27ac ChIP-seq heatmaps over embryonic enhancers. Data from Murphy et al, 2018 (sperm H3K4me1) and Zhang et al, 2018.

bioRxiv preprint doi: https://doi.org/10.1101/2024.08.26.609713; this version posted August 26, 2024. The copyright holder for this preprint (which was not certified by peer review) is the author/funder, who has granted bioRxiv a license to display the preprint in perpetuity. It is made available under aCC-BY 4.0 International license.

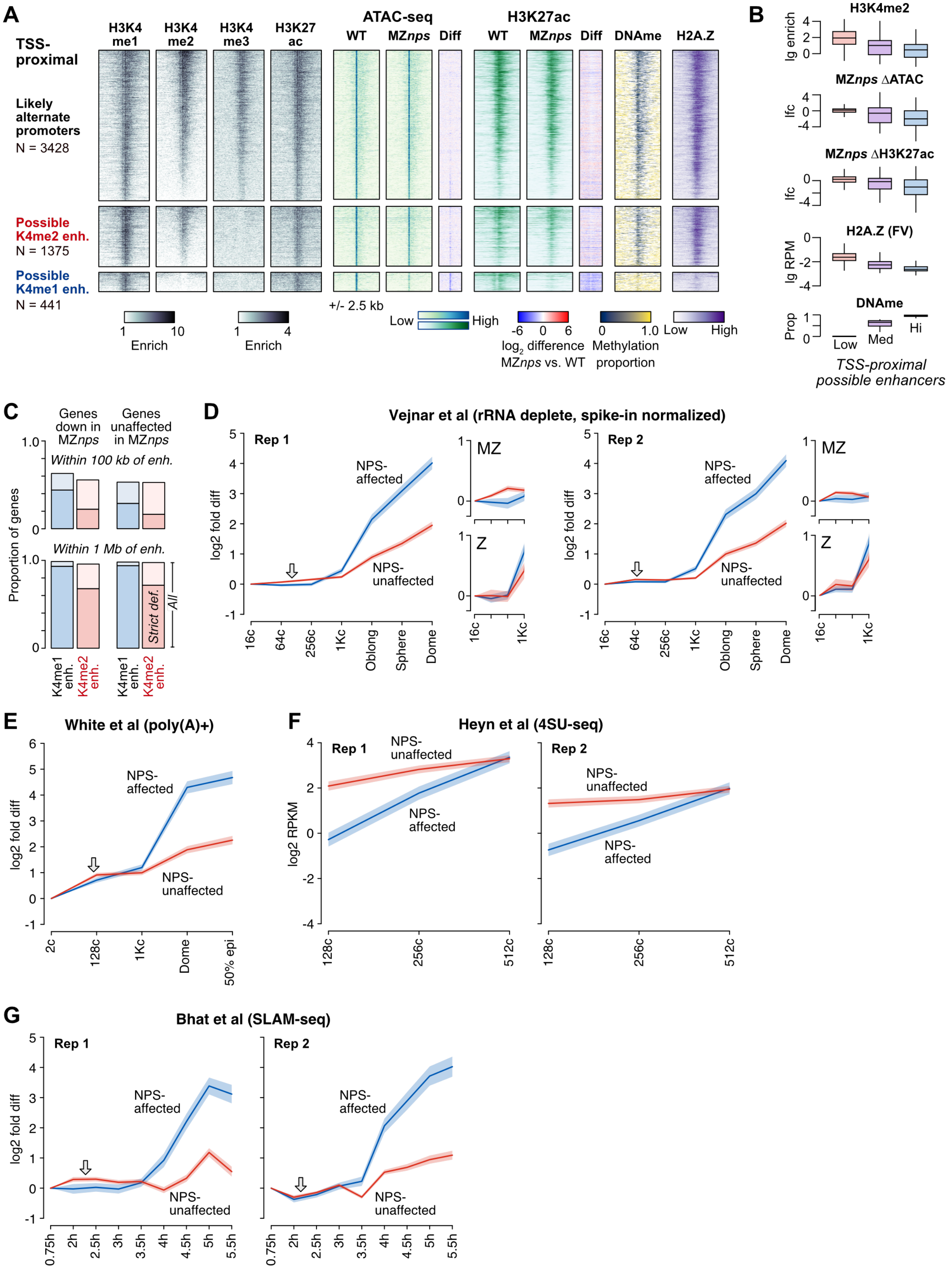

bioRxiv preprint doi: https://doi.org/10.1101/2024.08.26.609713; this version posted August 26, 2024. The copyright holder for this preprint (which was not certified by peer review) is the author/funder, who has granted bioRxiv a license to display the preprint in perpetuity. It is made available under aCC-BY 4.0 International license.

Supplementary Figure 5. Enhancer association with zygotic genes. (A) Expanded enhancer annotations: similar to Fig. 4, heatmaps of chromatin features over TSS-proximal elements excluded from the main enhancer analysis (<2 kb from, but not overlapping, any TSS, regardless if there is evidence for zygotic expression). Elements with H3K4me3 enrichment (top) are considered to be alternate promoters. The remaining elements segregate into possible H3K4me2 enhancers (middle group) and possible H3K4me1 enhancers (bottom group). Reporter assays suggest that such promoter-proximal regions can function as enhancers, despite the ambiguity in annotating them as such (Supp. Fig S3D). **(B)** Boxplots similar to Fig 4E summarizing the correlated chromatin features for TSS-proximal possible enhancers, which likewise segregate into hypermethylated, NPS-dependent, exclusively H3K4me1-marked enhancers and hypomethylated, non NPS-dependent, H3K4me2-marked enhancers. **(C)** Bar plots showing proportion of genes with 100 kb (top) or 1 Mb (bottom) of H3K4me1 enhancers (blue bars) and H3K4me2 enhancers (pink bars). Darker shaded region of each bar represents proportions limited to only strictly defined enhancers (TSS distal and <1.25-fold or ≥3-fold enriched for H3K4me2 for H3K4me1 and H3K4me2 enhancers, respectively). Lighter shaded regions include TSS-proximal elements. **(D-G)** Plots of average wild-type RNA-seq $\log_2$ fold increase over time as in Fig. 5C. MZ = maternal-zygotic genes only, Z = strictly zygotic genes only. Data are from Vejnar et al, 2019, White et al, 2017, Heyn et al, 2014, and Bhat et al, 2023.

bioRxiv preprint doi: https://doi.org/10.1101/2024.08.26.609713; this version posted August 26, 2024. The copyright holder for this preprint (which was not certified by peer review) is the author/funder, who has granted bioRxiv a license to display the preprint in perpetuity. It is made available under aCC-BY 4.0 International license.

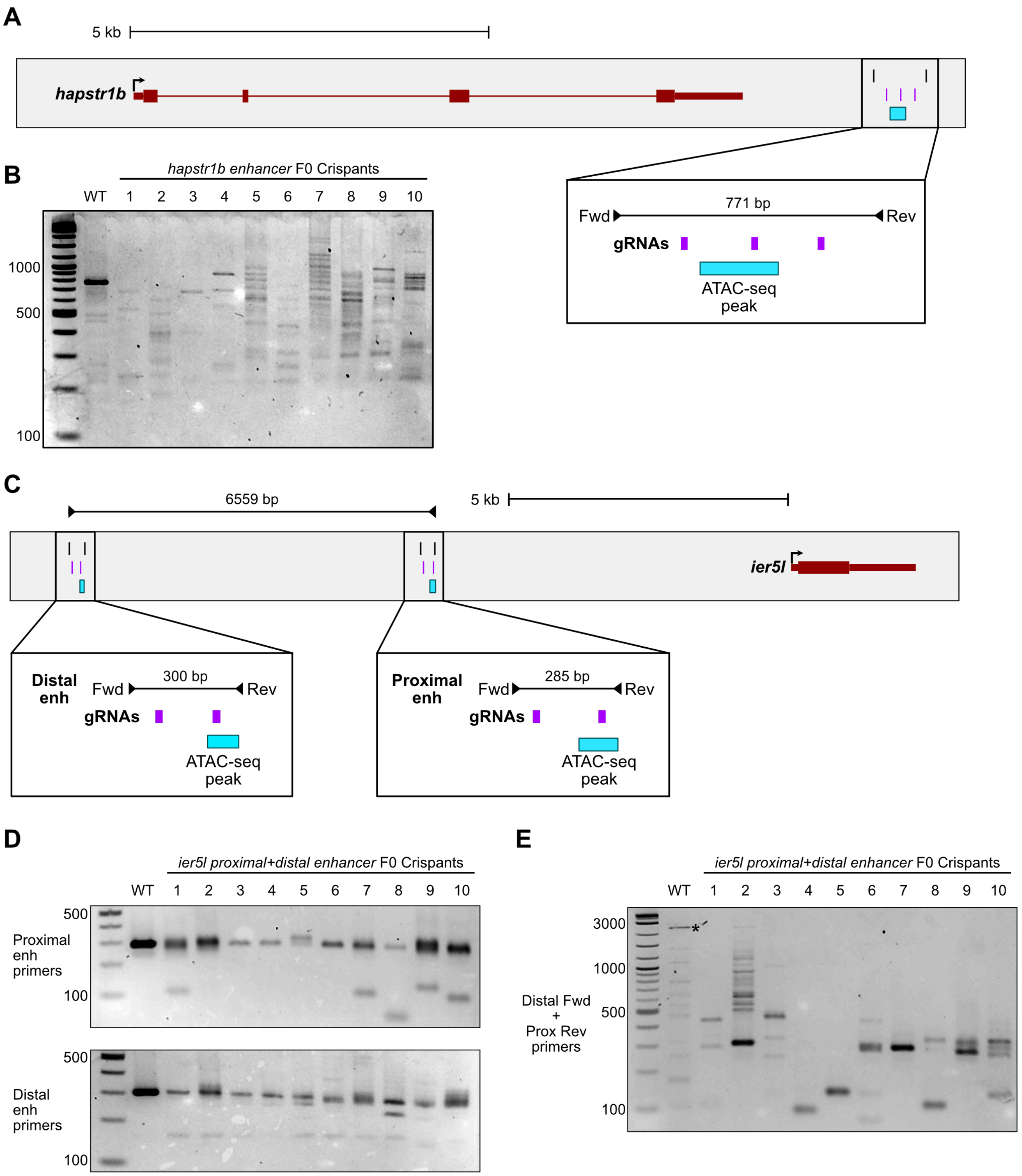

Supplementary Figure 6. Genotyping F0 enhancer crispants. (A) *hapstr1b* locus showing the ATAC-seq open region, CRISPR guide RNA target sites, and genotyping primers at the predicted downstream enhancer region. **(B)** Genotyping gel for single embryos at 32 hours post fertilization. Lane 1 = NEB 1kb Plus ladder, lane 2 = wild-type, lanes 3-12 = embryos injected with a pool of Cas9 complexed with each *hapstr1b* enhancer guide RNA. **(C)** *ier5l* locus showing two upstream predicted enhancers annotated as in (A). **(D)** Genotyping gels for single embryos using primers to detect lesions in the proximal enhancer (top) and distal enhancer (bottom). Crispants were injected with a pool for all gRNAs targeting both enhancers. Gel configuration similar to (B). **(E)** Genotyping gels for the same embryos as in (D) to detect large deletions spanning the two *ier5l* enhancers. The wild-type product (6559 bp) should not efficiently amplify under the PCR conditions used. Bands appearing in the wild-type lane are likely off-target products (asterisk).

bioRxiv preprint doi: https://doi.org/10.1101/2024.08.26.609713; this version posted August 26, 2024. The copyright holder for this preprint (which was not certified by peer review) is the author/funder, who has granted bioRxiv a license to display the preprint in perpetuity. It is made available under aCC-BY 4.0 International license.

Supplementary Table 1. CUT&RUN samples generated in this study

Supplementary Table 2. Regulatory regions defined in this study

Supplementary Table 3. Enhancer reporters

Supplementary Table 4. Sources of public data used
